# Bifurcation and Pattern Symmetry Selection in Reaction-Diffusion Systems with Kinetic Anisotropy

**DOI:** 10.1038/s41598-019-44303-2

**Published:** 2019-05-24

**Authors:** Yipeng Gao, Yongfeng Zhang, Daniel Schwen, Chao Jiang, Jian Gan

**Affiliations:** Idaho National Laboratory (INL), Idaho Falls, ID 83415 USA

**Keywords:** Coarse-grained models, Applied physics, Phase transitions and critical phenomena

## Abstract

Ordering and self-organization are critical in determining the dynamics of reaction-diffusion systems. Here we show a unique pattern formation mechanism, dictated by the coupling of thermodynamic instability and kinetic anisotropy. Intrinsically different from the physical origin of Turing instability and patterning, the ordered patterns we obtained are caused by the interplay of the instability from uphill diffusion, the symmetry breaking from anisotropic diffusion, and the reactions. To understand the formation of the void/gas bubble superlattices in crystals under irradiation, we establish a general theoretical framework to predict the symmetry selection of superlattice structures associated with anisotropic diffusion. Through analytical study and phase field simulations, we found that the symmetry of a superlattice is determined by the coupling of diffusion anisotropy and the reaction rate, which indicates a new type of bifurcation phenomenon. Our discovery suggests a means for designing target experiments to tailor different microstructural patterns.

## Introduction

Ordering and self-organization in reaction-diffusion systems are of great significance in determining the ordered patterns in chemistry, physics and biology^1–7^. Pioneered by Alan Turing, the mathematical description of reaction-diffusion systems are well established, and it has been recognized that the Turing instability originates from the reaction kinetics and the ordering is dictated by the breaking of continuous translational symmetry (i.e., the loss of homogeneity)^8,9^. However, the studies on another type of patterning mechanism in reaction-diffusion systems are limited, which originates from thermodynamic instability^10,11^ and the breaking of point symmetry (e.g., rotational or mirror symmetry)^12^. In physics, the self-organizations induced by thermodynamic instability and the breaking of point symmetry are widely observed in systems without reactions, e.g., multi-domain patterning in second-order ferroelectric and ferromagnetic phase transitions, which is dominated by long-range electric/magnetic interactions^13–16^. In those cases, long-range interactions play a critical role in pattern formation. In parallel, if local reactions are considered instead of non-local interactions, one should expect a new type of patterning mechanism in reaction-diffusion systems. Here we report a unique self-organization mechanism to understand the formation of void/gas bubble superlattices in crystals, which originates from the interplay of thermodynamic instability, diffusion anisotropy and reaction kinetics.

Literally, the dynamics of reaction-diffusion systems is dictated by the coupling of reaction and diffusion. Most of the previous studies focus on different kinds of reactions, while diffusivities are usually taken as positive (i.e., down-hill diffusion) and isotropic for simplicity^17,18^. In such cases, local reactions dominate the instability and the breaking of translational symmetry, while diffusion plays a secondary role on the ordering process. However, we cannot ignore another possibility that diffusion plays a dominate role over local reactions, when the diffusion flux is against concentration gradient (i.e., up-hill diffusion) and/or the diffusivity is anisotropic. In physics, up-hill diffusion could arise from thermodynamic instability^19,20^, while anisotropy suggests the breaking of point symmetry^21^. Given the above two fundamental pieces, instability and ordering could be realized even with a very simple reaction. Note that the reaction term in this case should not follow the constraint in Turing instability^18^, since it does not directly contribute to the instability and ordering. However, additional phenomena associated with local reactions could occur, e.g., bifurcation^17,22^, which might affect the symmetry selection of ordered patterns. In general, the bifurcation associated symmetry breaking could lead to diversified types of self-organized patterns. In real material systems, reaction-diffusion with the coupling of thermodynamic instability and breaking of point of symmetry can be found in crystals under irradiation. In particular, self-organized void/gas bubble superlattices have been widely observed in a large number of metals and alloys under irradiation^23–27^. In those systems, the reaction and diffusion of point defects are coupled with void formation and 1-dimensional interstitial diffusion, which result in diversified types of void/gas bubble superlattices as reported in the literature^28–30^.

In this paper, we investigate the ordering and self-organization in a reaction-diffusion system with thermodynamic instability and kinetic anisotropy, through a combination of analytical study and phase field simulations. We establish a general theoretical framework to predict the symmetry of superlattices associated with anisotropic diffusion. In particular, we demonstrate a unique formation mechanism of superlattice structures dictated by the interplay of diffusion anisotropy and local reactions. For a fixed type of anisotropic diffusivity, reaction rate works as a bifurcation parameter that could lead to different superlattice symmetries. Our discovery suggests a new way to design and control the symmetry of void/gas bubble superlattices in solid crystals under irradiation.

## Analytic Study of Reaction-Diffusion Systems with Kinetic Anisotropy

Without the loss of generality, our analytical study starts with a generic description of reaction-diffusion systems, followed by specific anisotropies incorporated into phase field modeling and simulations. Mathematically, the dynamics of reaction-diffusion systems is described by partial differential equations including two kinds of terms, i.e., diffusion terms and reaction terms. In this study, we focus on the instability and ordering caused by different diffusion terms. Here we consider two ways to modify the diffusion terms, by adopting Cahn-Hilliard type diffusion^31^ and anisotropic diffusion. The former could introduce thermodynamic instability and up-hill diffusion, while the latter suggests a breaking of point symmetry. In a reaction-diffusion system, we consider two components, the diffusion kinetics of which are dominated by Cahn-Hilliard equation and anisotropic diffusion, respectively. A simple reaction term, i.e., annihilation, is considered to couple the evolutions of the two components. Source terms are also considered to balance the annihilation. The following equations are employed,

1 $$\frac{\partial X}{\partial t}=\nabla \cdot M\nabla \frac{\delta F}{\delta X}+{P}_{X}-\sum _{i\mathrm{=1}}^{n}\,KX{Y}_{i}$$

2 $$\frac{\partial {Y}_{i}}{\partial t}=\nabla \cdot {{\bf{D}}}_{i}\nabla {Y}_{i}+{P}_{i}-KX{Y}_{i},\,i=\mathrm{1,2},\mathrm{...},n$$

*X* and *Y*_*i*_ are the concentrations of the two components. The diffusion of *X* is Cahn-Hilliard type, with a mobility of *M*. The diffusion of *Y*_*i*_ is anisotropy, and there could be *n*-th types of *Y*_*i*_ with different diffusivity D_*i*_. *P*_*X*_ and *P*_*i*_ are the source terms for *X* and *Y*_*i*_, respectively. *F* is the total free energy of the system. *K* is the reaction rate for the annihilation between *X* and *Y*.

*F* is the total free energy of a non-uniform system, which can be described as below, with the gradient term incorporated.

3 $$F=\int \,[f(X)+\frac{1}{2}\kappa {(\nabla X)}^{2}]{d}^{3}r$$

*f* is the bulk free energy density, and *κ* is the coefficient of gradient energy, which captures the energetic penalty of inhomogeneity.

Here we consider an anisotropic diffusivity along a 1-dimensional (1D) direction, which can be represented in a tensor form.

4 $${{\bf{D}}}_{i}={D}_{0}\cdot {{\bf{m}}}^{i}\otimes {{\bf{m}}}^{i}$$

where **m**^*i*^ is a unit vector describing the direction of the 1D diffusion for *Y*_*i*_, and *D*_0_ is the 1D diffusivity along **m**^*i*^ direction. ⊗ is the diadic product operator.

For an analytic study, we consider a linear approximation of the reaction term.

5 $$KX{Y}_{i}=K(\bar{X}+\delta X)({\bar{Y}}_{i}+\delta {Y}_{i})\approx K\bar{X}{\bar{Y}}_{i}+K\bar{X}\delta {Y}_{i}+K{\bar{Y}}_{i}\delta X$$

$\bar{X}$ and ${\bar{Y}}_{i}$ are the averaged values of *X* and *Y*_*i*_, respectively, which are spatially-independent. As a result, we can express $X=\bar{X}+\delta X$ and ${Y}_{i}={\bar{Y}}_{i}+\delta {Y}_{i}$, where *δX* and *δY*_*i*_ are perturbations. The above approximation is valid when the pertubations are relatively small (comparing with the averaged values), which corresponds to the initial stage of modulation. As a result, Eqs 1 and 2 can be represented in Fourier space.

6 $$\frac{\partial \tilde{X}}{\partial t}={G}_{11}({\bf{k}})\tilde{X}+\sum _{i=1}^{n}\,{G}_{1,i+1}({\bf{k}})\cdot {\tilde{Y}}_{i}$$

7 $$\frac{\partial {\tilde{Y}}_{i}}{\partial t}={G}_{i+\mathrm{1,1}}({\bf{k}})\cdot \tilde{X}+{G}_{i+\mathrm{1,}i+1}({\bf{k}})\cdot {\tilde{Y}}_{i},\,i=\mathrm{1,2},\mathrm{...},n$$

$\tilde{X}$ and ${\tilde{Y}}_{i}$ are the Fourier transforms of *X* and *Y*_*i*_, respectively. Excluding **k** = 0, $\tilde{X}$ and ${\tilde{Y}}_{i}$ are also the Fourier transforms of *δX* and *δY*_*i*_. *G* is the coefficient matrix (a square matrix of *n* + 1 order) of the above partial differential equations. In the index of *G*, subscript 1 indicates *X*, and $2 \sim (n+1)$ indicate *n* types of *Y*_*i*_. *G* is a function of the wave vector *k*, as well as all the above thermodynamic and kinetic parameters (i.e., *F*, $\bar{X}$, ${\bar{Y}}_{i}$, *M*, *D*, *K*).

Assuming the first developed wave is **k**_*c*_. The critical conditions in determining **k**_*c*_ should include the following two equations,

8 $$det[G({{\bf{k}}}_{c})]=0$$

9 $$\frac{d\{det[G({\bf{k}})]\}}{d{\bf{k}}}{|}_{{\bf{k}}={{\bf{k}}}_{c}}=0$$

Note that **k** is a wave vector having both magnitude k and direction $\hat{k}={\bf{k}}/|{\bf{k}}|$, which determine the characteristic wavelength and symmetry of a superlattice, respectively. In order to simplify the above equation, we consider *n* equivalent types of *Y*_*i*_ (in terms of their diffusion anisotropy), with ${\bar{Y}}_{i}=\bar{Y}$. As a result, we can separate the length and direction of **k** in *det*[*G*(**k**)].

10 $${\det }[G({\bf{k}})]={a}_{3}(k){(-1)}^{n}[{a}_{1}(k)+\sum _{i=1}^{n}\,\frac{1}{{({{\bf{m}}}^{i}\cdot \hat{k})}^{2}+{a}_{2}(k)}]\prod _{j=1}^{n}\,[{({{\bf{m}}}^{j}\cdot \hat{k})}^{2}+{a}_{2}(k)]$$

where

11 $${a}_{1}=\frac{{D}_{0}{k}^{2}(\,-\,M^{\prime\prime} {k}^{2}-M\kappa {k}^{4}-nK\bar{Y})}{{K}^{2}\bar{X}\bar{Y}}$$

12 $${a}_{2}=\frac{K\bar{X}}{{D}_{0}{k}^{2}}$$

13 $${a}_{3}={K}^{2}\bar{X}\bar{Y}{({D}_{0}{k}^{2})}^{n-1}$$

*a*_1_, *a*_2_ and *a*_3_ are parameters depending on the magnitude of  **k** only, while other part of Eq. 10 depends on the direction $\hat{k}$ only. The solution of **k**_*c*_ corresponds to a minimum of *det*(*G*) if *n* is odd, while it corresponds to a maximum if *n* is even. In the following discussion, we take an example of the 1D diffusion along equivalent $(\frac{\sqrt{\mathrm{(3)}}}{3}\frac{\sqrt{\mathrm{(3)}}}{3}\frac{\sqrt{\mathrm{(3)}}}{3})$ directions in a Cartesian coordinate system, i.e., ${{\bf{m}}}^{i}=(\frac{\sqrt{\mathrm{(3)}}}{3}\frac{\sqrt{\mathrm{(3)}}}{3}\frac{\sqrt{\mathrm{(3)}}}{3})$, $(\frac{-\sqrt{\mathrm{(3)}}}{3}\frac{\sqrt{\mathrm{(3)}}}{3}\frac{\sqrt{\mathrm{(3)}}}{3})$, $(\frac{\sqrt{\mathrm{(3)}}}{3}\frac{-\sqrt{\mathrm{(3)}}}{3}\frac{\sqrt{\mathrm{(3)}}}{3})$, $(\frac{\sqrt{\mathrm{(3)}}}{3}\frac{\sqrt{\mathrm{(3)}}}{3}\frac{-\sqrt{\mathrm{(3)}}}{3})$, with *n* = 4. For any given values of *a*_1_, *a*_2_ and *a*_3_, we can determine the critical ${\hat{k}}_{c}$ direction that maximizes *det*[*G*(*k*)]. In the above equation, *a*_1_ and *a*_2_ are parameters being coupled with such a maximization process, while *a*_3_ is decoupled. As a result, a numerical calculation to maximize *det*(*G*) (with respect to $\hat{k}$) can be performed for a given set of *a*_1_ and *a*_2_.

**Figure 1:**
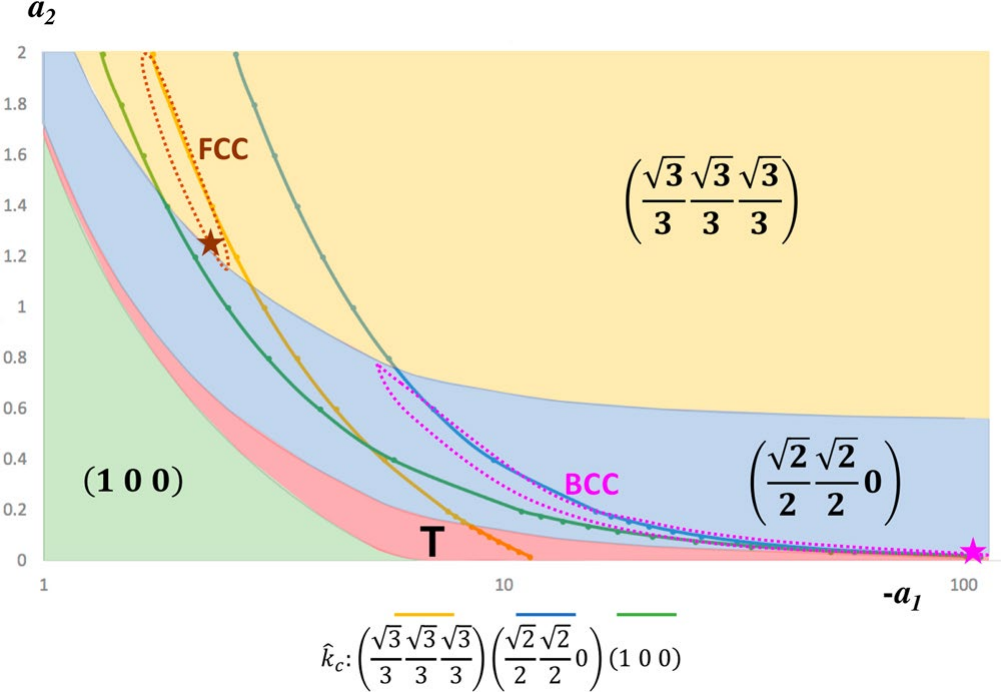
Symmetry selection of superlattices associated with $(\frac{\sqrt{\mathrm{(3)}}}{3}\frac{\sqrt{\mathrm{(3)}}}{3}\frac{\sqrt{\mathrm{(3)}}}{3})$ type 1D diffusion. The dominant concentration waves in different colored regions are distinctive. The critical condition for the development of a concentration wave is described by solid lines (different types of wave are suggested by different colors). The interplay of the two conditions implies the formation of the superlattices, i.e., the blue line in the blue region suggests BCC superlattice formation, and the yellow line in the yellow region suggests FCC superlattice formation. Numerical simulations are performed at the star points.

For 1D diffusion of *Y*_*i*_ along equivalent $(\frac{\sqrt{\mathrm{(3)}}}{3}\frac{\sqrt{\mathrm{(3)}}}{3}\frac{\sqrt{\mathrm{(3)}}}{3})$ directions, since *a*_1_ is negative near the critical point, we use −*a*_1_ in our following discussions for convenience. The symmetry selection of superlattice associated with $(\frac{\sqrt{\mathrm{(3)}}}{3}\frac{\sqrt{\mathrm{(3)}}}{3}\frac{\sqrt{\mathrm{(3)}}}{3})$ type 1D diffusion is shown in Fig. 1. The horizontal axis is −*a*_1_ with logarithmic scale, while the vertical axis is *a*_2_. Depending on the choice of −*a*_1_ and *a*_2_, there are four distinctive regions, in which different **k** directions are dominant. When both −*a*_1_ and *a*_2_ are large, $(\frac{\sqrt{\mathrm{(3)}}}{3}\frac{\sqrt{\mathrm{(3)}}}{3}\frac{\sqrt{\mathrm{(3)}}}{3})$ is the preferred **k** direction (yellow region). When both −*a*_1_ and *a*_2_ are small, (100) is the preferred **k** direction (green region). Between the yellow and green regions, there is a region preferring the $(\frac{\sqrt{\mathrm{(2)}}}{2}\frac{\sqrt{\mathrm{(2)}}}{2}0)$ direction (blue region). There is also a slim transition region (red region noted by T) between the blue and green regions, with the preferred **k** direction changing gradually from $(\frac{\sqrt{\mathrm{(2)}}}{2}\frac{\sqrt{\mathrm{(2)}}}{2}0)$ to (100). Note that the four regions in Fig. 1 only illustrate the effects of *a*_1_ and *a*_2_ on the symmetry selection based on Eq. 9. The critical condition described by Eq. 8 should also be taken into account, which leads to another relation between *a*_1_ and *a*_2_.

14 $${a}_{1}=-\,\sum _{i\mathrm{=1}}^{n}\frac{1}{{({m}^{i}\cdot \hat{k})}^{2}+{a}_{2}}$$

Here *a*_1_ is a function of *a*_2_ for a given direction of k, which suggests a curve including all critical points. In Fig. 1, we plot three solid lines for ${\hat{k}}_{c}$ along $(\frac{\sqrt{\mathrm{(3)}}}{3}\frac{\sqrt{\mathrm{(3)}}}{3}\frac{\sqrt{\mathrm{(3)}}}{3})$ (yellow curve), $(\frac{\sqrt{\mathrm{(2)}}}{2}\frac{\sqrt{\mathrm{(2)}}}{2}0)$ (blue curve) and (100) (green curve), respectively. As a result, when a curve is located in a region with the same color, it suggests the formation of superlattice. In Fig. 1, there are two possible regions of superlattice formation. In the region circled by magenta dashed lines, the preferred **k** direction is $(\frac{\sqrt{\mathrm{(2)}}}{2}\frac{\sqrt{\mathrm{(2)}}}{2}0)$, which results in a face-centered cubic (FCC) reciprocal lattice and a body-centered cubic (BCC) real superlattice. In the region circled by brown dashed lines, the preferred **k** direction is $(\frac{\sqrt{\mathrm{(3)}}}{3}\frac{\sqrt{\mathrm{(3)}}}{3}\frac{\sqrt{\mathrm{(3)}}}{3})$, which results in a BCC reciprocal lattice and an FCC real superlattice. Note that superlattice could form when *a*_1_ is slightly larger than its critical value described by Eq. 14, which suggests that superlattice formation region should attach to the left of the critical curve.

Similar analysis can be applied to the systems with 1D diffusion along $(\frac{\sqrt{\mathrm{(2)}}}{2}\frac{\sqrt{\mathrm{(2)}}}{2}0)$ directions, i.e., $(\frac{\sqrt{\mathrm{(2)}}}{2}\frac{\sqrt{\mathrm{(2)}}}{2}0)$, $(\frac{-\sqrt{\mathrm{(2)}}}{2}\frac{\sqrt{\mathrm{(2)}}}{2}0)$, $(\frac{\sqrt{\mathrm{(2)}}}{2}0\frac{\sqrt{\mathrm{(2)}}}{2})$, $(\frac{-\sqrt{\mathrm{(2)}}}{2}0\frac{\sqrt{\mathrm{(2)}}}{2})$, $(0\frac{\sqrt{\mathrm{(2)}}}{2}\frac{\sqrt{\mathrm{(2)}}}{2})$, $(0\frac{-\sqrt{\mathrm{(2)}}}{2}\frac{\sqrt{\mathrm{(2)}}}{2})$, with *n* = 6. The preferred **k** direction is always $(\frac{\sqrt{\mathrm{(3)}}}{3}\frac{\sqrt{\mathrm{(3)}}}{3}\frac{\sqrt{\mathrm{(3)}}}{3})$ type (for any given *a*_1_ and *a*_2_ constrained by Eq. 14). As a result, the **k**_*c*_ vectors suggest a BCC reciprocal lattice and an FCC real superlattice. The mathematical expressions of *G* as well as detailed predictions of characteristic length and symmetry of superlattices are presented in Supporting Information, which are consistent with previous theoretical studies^32,33^.

## Phase Field Modeling and Simulations of the Formation of Self-Organized Superlattices in Crystals

**Figure 2:**
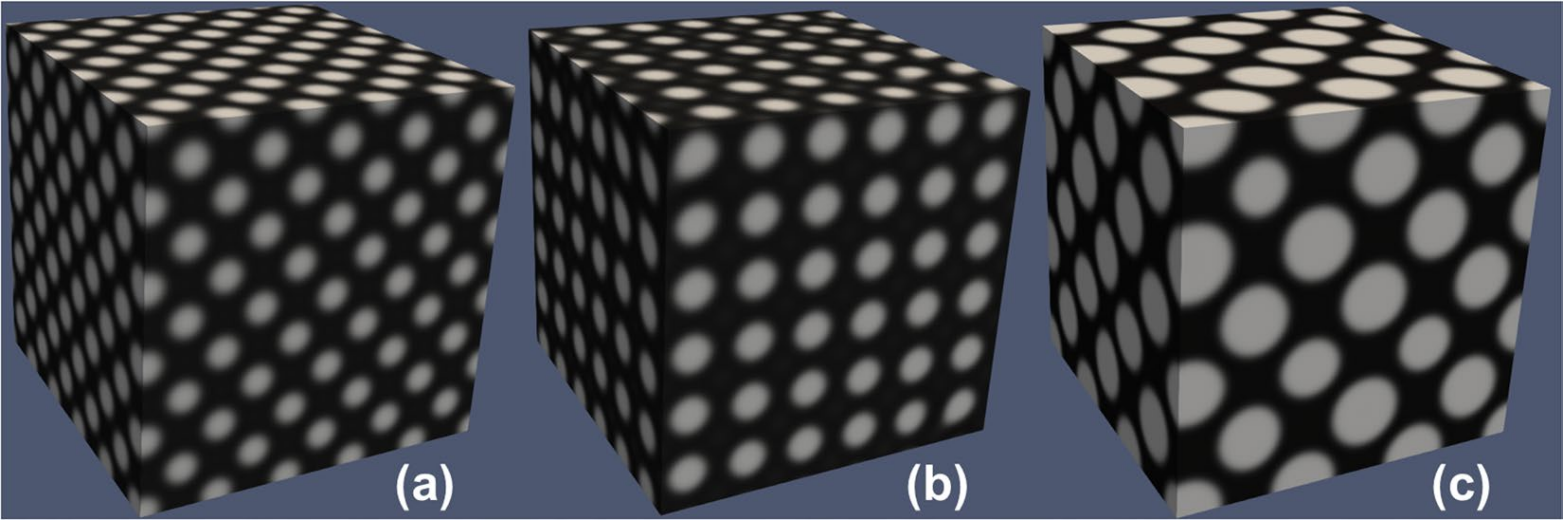
Phase field simulation results of superlattices in reaction-diffusion systems with kinetic anisotropy (white: *X*-rich, black: *X*-lean). (**a**) an FCC superlattice associated with $(\frac{\sqrt{\mathrm{(2)}}}{2}\frac{\sqrt{\mathrm{(2)}}}{2}0)$ type 1D diffusion; (**b**) a BCC superlattice associated with $(\frac{\sqrt{\mathrm{(3)}}}{3}\frac{\sqrt{\mathrm{(3)}}}{3}\frac{\sqrt{\mathrm{(3)}}}{3})$ type 1D diffusion; (**c**) an FCC superlattice associated with $(\frac{\sqrt{\mathrm{(3)}}}{3}\frac{\sqrt{\mathrm{(3)}}}{3}\frac{\sqrt{\mathrm{(3)}}}{3})$ type 1D diffusion.

We perform phase field modeling and simulations (in a 100 × 100 × 100 simulation cell)^34–36^ to validate our analytical predictions. Modeling details are presented in Supporting Information. By choosing different sets of *a*_1_ and *a*_2_, we obtain an FCC superlattice associated with $(\frac{\sqrt{\mathrm{(2)}}}{2}\frac{\sqrt{\mathrm{(2)}}}{2}0)$ type 1D diffusion (Fig. 2(a)), BCC and FCC superlattices associated with $(\frac{\sqrt{\mathrm{(3)}}}{3}\frac{\sqrt{\mathrm{(3)}}}{3}\frac{\sqrt{\mathrm{(3)}}}{3})$ type 1D diffusion (Fig. 2(b,c), corresponding to the magenta and brown stars in Fig. 1, respectively). The related parameters are listed in Table 1. As expected, the simulation results perfectly agree with our analytical predictions shown in Fig. 1.

**Table 1:** Parameters for phase field simulations.

Example	*a*_1_	*a*_2_	1D diffusion	Superlattice	Lattice constant
Figs 2(a), 3 and 4	−157.3	0.0101	〈110〉	FCC	16.0
Figs 2(b) and 5	−101.8	0.0105	〈111〉	BCC	13.3
Figs 2(c) and 6	−2.37	1.26	〈111〉	FCC	26.7

**Figure 3:**
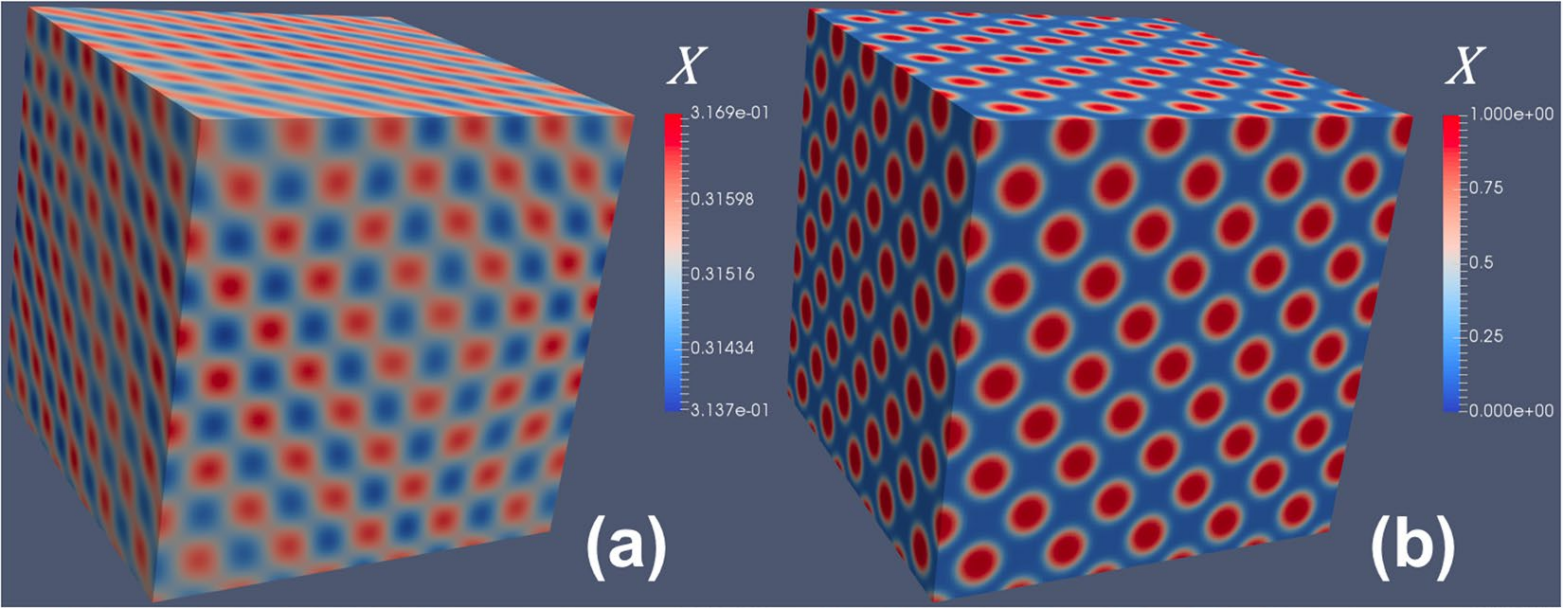
Simulation results of *X* concentration for an FCC superlattice associated with $(\frac{\sqrt{\mathrm{(2)}}}{2}\frac{\sqrt{\mathrm{(2)}}}{2}0)$ type 1D diffusion, corresponding to Fig. 2(a). (**a**) Initial stage: chessboard-like modulation; (**b**) final stage: FCC superlattice.

**Figure 4:**
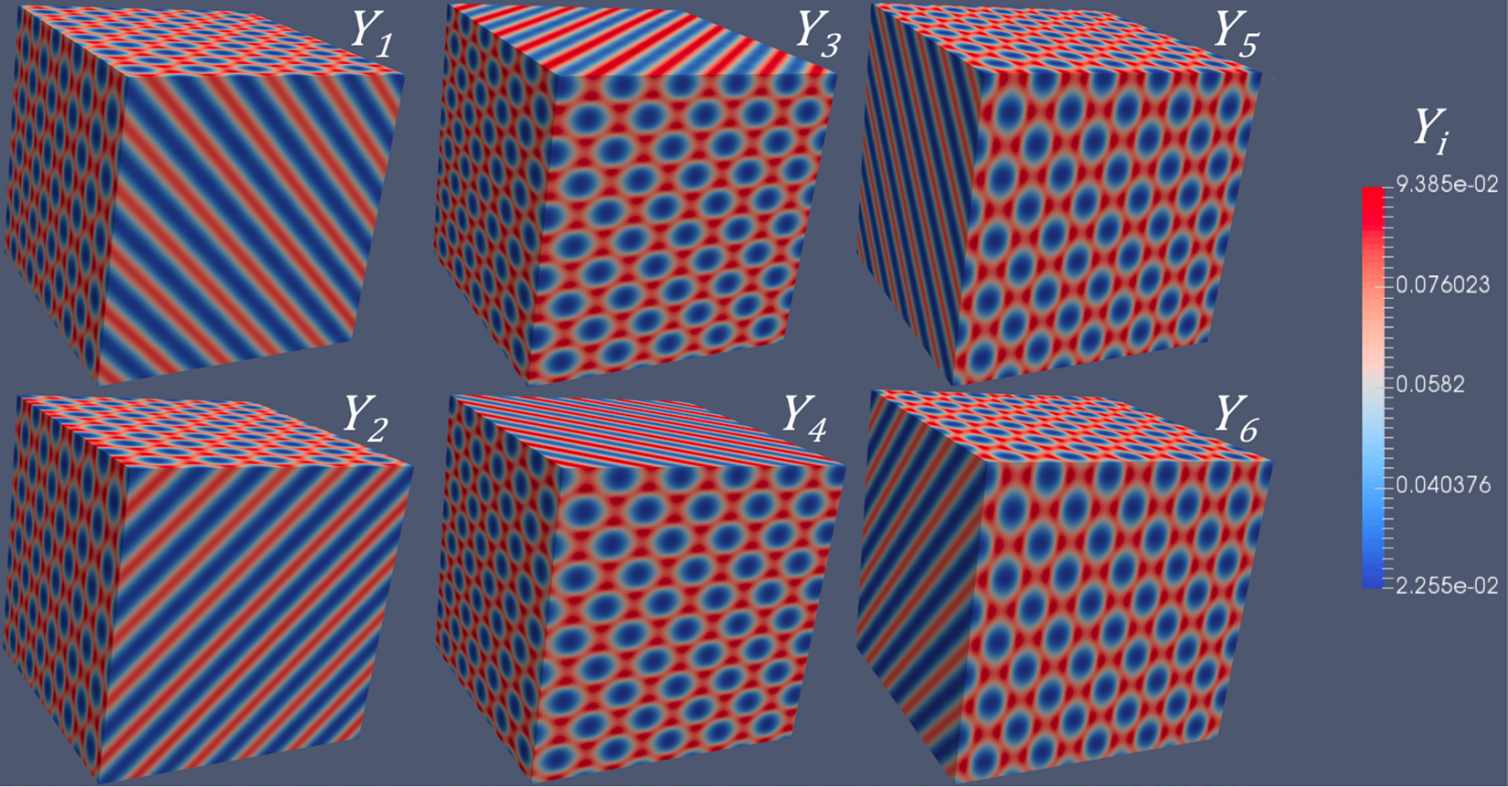
Simulation results of six types of *Y*_*i*_ concentrations for an FCC superlattice associated with $(\frac{\sqrt{\mathrm{(2)}}}{2}\frac{\sqrt{\mathrm{(2)}}}{2}0)$ type 1D diffusion, corresponding to Fig. 2(a).

To further understand the coupling between different components, we plot the concentrations of *X* in Fig. 3, for $(\frac{\sqrt{\mathrm{(2)}}}{2}\frac{\sqrt{\mathrm{(2)}}}{2}0)$ type of 1D diffusion. At the initial stage, a chessboard-like modulation of *X* is developed (Fig. 3(a)), which finally evolves to an FCC superlattice (Fig. 3(b)). When the superlattice forms, the concentrations of six types of *Y*_*i*_ are plotted in Fig. 4, which correspond to the six equivalent directions of $(\frac{\sqrt{\mathrm{(2)}}}{2}\frac{\sqrt{\mathrm{(2)}}}{2}0)$ type. It can be found that a 1D diffusion along [110] weakens the modulation along this [110]. The interplay of six types of *Y*_*i*_ modulations are coupled with concentration wave of *X*, which finally results in an FCC superlattice.

**Figure 5:**
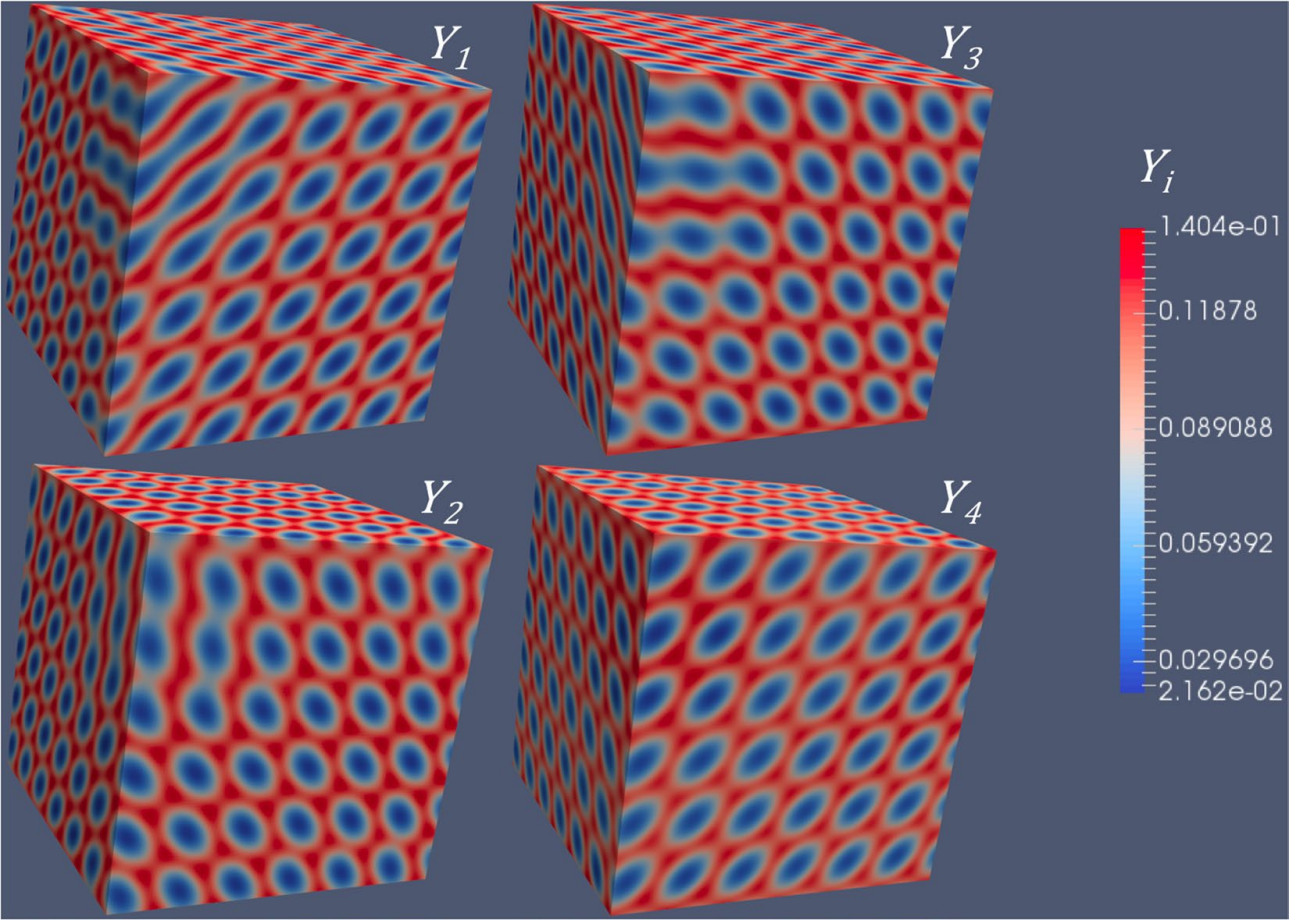
Simulation results of four types of *Y*_*i*_ concentrations for an BCC superlattice associated with $(\frac{\sqrt{\mathrm{(3)}}}{3}\frac{\sqrt{\mathrm{(3)}}}{3}\frac{\sqrt{\mathrm{(3)}}}{3})$ type 1D diffusion, corresponding to Fig. 2(b).

**Figure 6:**
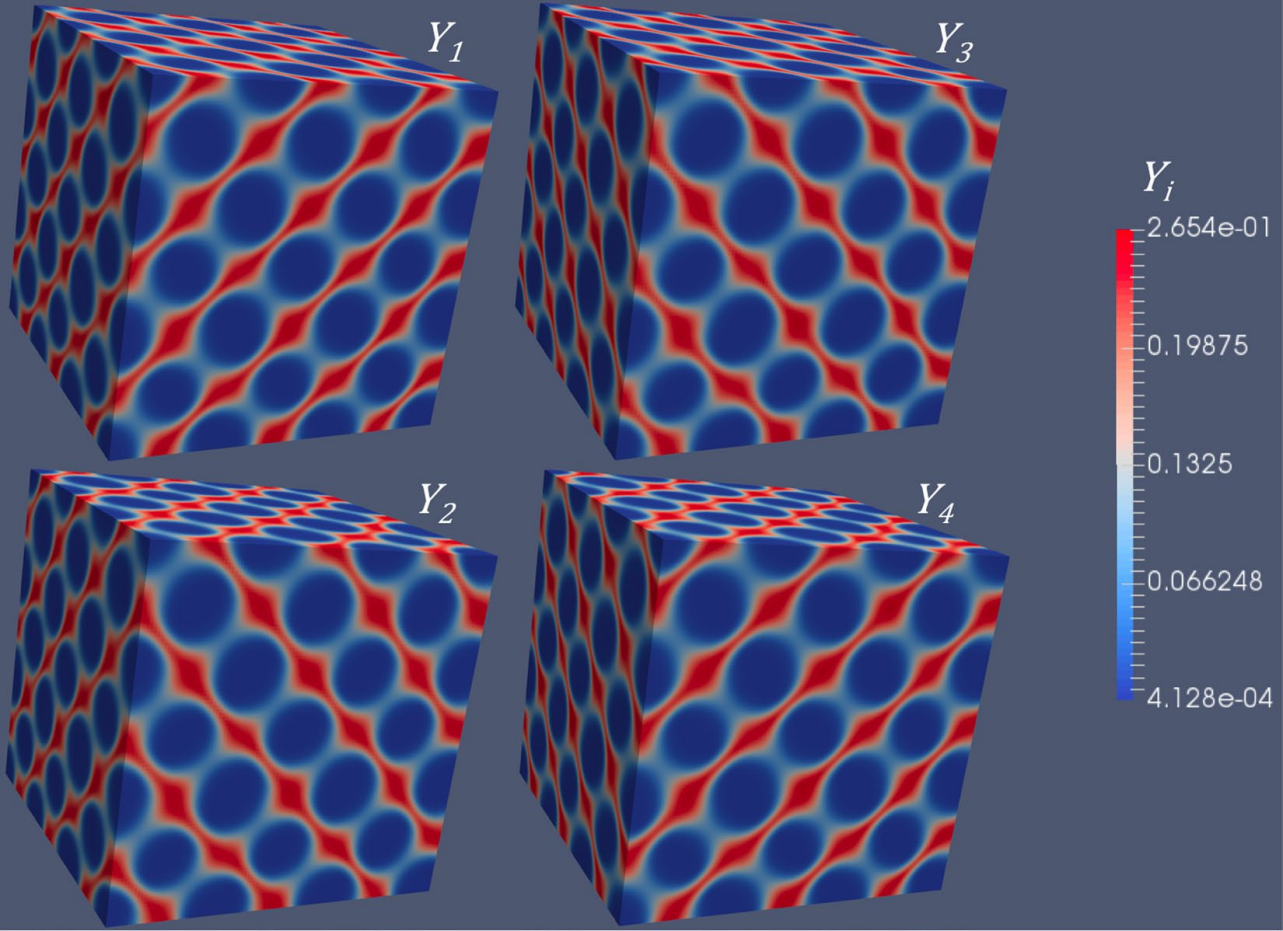
Simulation results of four types of *Y*_*i*_ concentrations for an FCC superlattice superlattice associated with $(\frac{\sqrt{\mathrm{(3)}}}{3}\frac{\sqrt{\mathrm{(3)}}}{3}\frac{\sqrt{\mathrm{(3)}}}{3})$ type 1D diffusion, corresponding to Fig. 2(c).

Similarly, the concentration modulations of four different types of *Y*_*i*_ are shown in Figs 5 and 6, for $(\frac{\sqrt{\mathrm{(3)}}}{3}\frac{\sqrt{\mathrm{(3)}}}{3}\frac{\sqrt{\mathrm{(3)}}}{3})$ type of 1D diffusion. According to our previous calculations, either BCC or FCC superlattice can form. The concentration modulations of ${Y}_{1} \sim {Y}_{4}$ associated with a BCC superlattice is shown in Fig. 5, while those associated with an FCC superlattice is shown in Fig. 6. Comparing the figures in Fig. 5, we can clearly identify the difference in the concentration modulations of *Y*_*i*_, i.e., different symmetry. Note that the symmetry of modulations originates from the anisotropic diffusivities of *Y*_*i*_. Without changing the anisotropy, i.e., $(\frac{\sqrt{\mathrm{(3)}}}{3}\frac{\sqrt{\mathrm{(3)}}}{3}\frac{\sqrt{\mathrm{(3)}}}{3})$ type of diffusion, we can get completely different symmetries, which is caused by the change of scalar parameters (i.e., *a*_1_ and *a*_2_). For simplicity, we only consider one independent variable, *a*_2_, since *a*_1_ is a function of *a*_2_ at the critical condition (Eq. 14).

In fact, *a*_2_ reflects a competition between reaction (*K*) and diffusion (*D*_0_), and it is also influenced by the critical wavelength (*λ*_*c*_ = 2*π*/**k**_*c*_) and the critical concentration $(\bar{X})$ when the homogeneous system starts to lose stability. In reaction-diffusion systems, it is well known that the change of reaction/diffusion rate could lead to bifurcation phenomena. Here *a*_2_ can be taken as a bifurcation parameter. Note that *a*_2_ is a scalar without any symmetry information. However, the change of *a*_2_ switches the symmetry of a superlattice. Such a unique bifurcation phenomenon could provide a new insight into the formation of void and gas bubble superlattices in crystals induced by irradiation.

The reaction-diffusion system we discussed above could correspond to a solid crystal under irradiation. For example, a lattice atom can be knocked out by implanted particles (e.g., ions or neutrons), which generates a frenkel pair, i.e., a vacancy and an interstitial atom^37^. In crystals, vacancies and interstitials (also called self-interstitial atoms, SIAs) are point defects of opposite nature (e.g., defect and anti-defect), which can disappear through recombination, i.e., annihilation. The concentrations of vacancies and interstitials can be described by *X* and *Y*. In the literature, it has been reported that SIAs and their clusters (e.g., interstitial loops) usually diffuse along a 1D crystallographic direction because of the lattice discreteness of crystals^38,39^. As a result, Eqs 1 and 2 capture the production, reaction and evolutions of defects in crystals under irradiation, in which the rate theory for production and reaction kinetics^40^ and the Cahn-Hillard approach for the phase separation description of void formation^19,41^ are coupled together. Thermodynamic instability are described through the formulation of *F* in Eq. 1, which leads the accumulation of vacancies and the formation of voids^28–30,34,42,43^. The types of SIAs depend on the symmetry of host crystals. For example, in a BCC crystal, if the SIAs diffuse in 1D along the close-packed directions 〈111〉, there are four types of SIAs (*n* = 4), which diffuse along $\mathrm{[111],}\,\mathrm{[11}\bar{1}],\,\mathrm{[1}\bar{1}\mathrm{1],}\,[\bar{1}\mathrm{11]}$, respectively. In an FCC crystal, if the SIAs diffuse in 1D along 〈110〉, there are six types of SIAs or clusters (*n* = 6), which diffuse along $\mathrm{[110],}\,\mathrm{[011],}\,\mathrm{[101],}\,\mathrm{[1}\bar{1}\mathrm{0],}\,\mathrm{[01}\bar{1}],\,[\bar{1}\mathrm{01]}$, respectively. Note that we treat the SIAs diffusing along different directions as distinct types of *Y*_*i*_, rather than treating them as a single type with equal diffusivity along several crystallographically equvalent directions. Theoretically, diffusivity is a second-rank tensor, which has to be isotropic in cubic crystal^21^. For example, if an interstitial atom jumps along four 〈111〉 directions with equal probability in one step, it essentially diffuses isotropically after several steps. Furthermore, it is possible that an interstitial atom does not jump strictly along one direction in reality, i.e., it may change its direction. In such a case, reaction terms among different *Y*_*i*_ should be included. However, we do not consider those terms due to analytical complexity.

As suggested by experimental observations, only FCC superlattice can form in FCC host crystals^27,44,45^, while either BCC or FCC superlattice can form in BCC host crystals^46–48^, which agree with our theoretical predictions. As suggested by our analyses, the symmetry selection of superlattices is dictated by *a*_1_ and *a*_2_, which are determined by the interplay of thermodynamic/kinetic properties of the material systems and radiation conditions (details can be found in Supplemental Information). Void/gas bubble superlattices widely observed in experiments also suggest the stability of superlattice under irradiation. In our phase field simulations, we obtain stable superlattices upon further relaxation, which is caused by the dynamic equilibrium between defect generation and recombination. Without irradiation (e.g., the reaction terms), the superlattice is not thermodynamically stable. Coarsening can occur driven by the minimization of surface energy. Such coarsening will be very slow for order superlattices. The study of superlattice stability is beyond our symmetry analyses in this paper, which will be conducted in future work.

## Conclusion

Through analytical study and phase field simulations, we establish a general theoretical framework to predict the ordering and self-organization in reaction-diffusion systems with thermodynamic instability and kinetic anisotropy. It is found that the pattern symmetry is determined by the interplay of anisotropic diffusions and local reactions. A new bifurcation phenomenon is demonstrated, which provides a new insight into the formation mechanism of irradiation-induced void/gas bubble superlattices in crystals.

## Supplementary information

Supplemental Info

SI LaTeX File

## References

[1] Grindrod, P. *The theory and applications of reaction-diffusion equations: patterns and waves*. Clarendon Press (1996).

[2] Kondo S, Miura T (2010). Reaction-diffusion model as a framework for understanding biological pattern formation. science.

[3] Bisquert J (2002). Theory of the impedance of electron diffusion and recombination in a thin layer. The Journal of Physical Chemistry B.

[4] Vanag VK, Epstein IR (2009). Cross-diffusion and pattern formation in reaction-diffusion systems. Physical Chemistry Chemical Physics.

[5] Itatani M, Fang Q, Unoura K, Nabika H (2018). Role of nuclei in liesegang pattern formation: Insights from experiment and reaction-diffusion simulation. The Journal of Physical Chemistry C.

[6] Liu Q (2014). Pattern formation at multiple spatial scales drives the resilience of mussel bed ecosystems. Nature communications.

[7] Vandegehuchte BD, Choudhury IR, Thybaut JW, Martens JA, Marin GB (2014). Integrated stefan-maxwell, mean field, and single-event microkinetic methodology for simultaneous diffusion and reaction inside microporous materials. The Journal of Physical Chemistry C.

[8] Turing AM (1952). The chemical basis of morphogenesis. Phil. Trans. R. Soc. Lond. B.

[9] Reinitz J (2012). Turing centenary: pattern formation. Nature.

[10] Holland PM, Rubingh DN (1983). Nonideal multicomponent mixed micelle model. The Journal of Physical Chemistry.

[11] Carati D, Lefever R (1997). Chemical freezing of phase separation in immiscible binary mixtures. Physical Review E.

[12] Busiello DM, Planchon G, Asllani M, Carletti T, Fanelli D (2015). Pattern formation for reactive species undergoing anisotropic diffusion. The European Physical Journal B.

[13] Chen L-Q, Khachaturyan AG (1993). Dynamics of simultaneous ordering and phase separation and effect of long-range coulomb interactions. Phys. Rev. Lett..

[14] Chae SC (2010). Self-organization, condensation, and annihilation of topological vortices and antivortices in a multiferroic. Proceedings of the National Academy of Sciences.

[15] Donnio B, García-Vázquez P, Gallani J-L, Guillon D, Terazzi E (2007). Dendronized ferromagnetic gold nanoparticles self-organized in a thermotropic cubic phase. Advanced Materials.

[16] Scholl, E. Nonequilibrium phase transitions in semiconductors: self-organization induced by generation and recombination processes. *Springer Berlin-New York* (1987).

[17] Callahan TK, Knobloch E (1999). Pattern formation in three-dimensional reaction–diffusion systems. Physica D: Nonlinear Phenomena.

[18] Satnoianu RA, Menzinger M, Maini PK (2000). Turing instabilities in general systems. Journal of mathematical biology.

[19] Cahn JW (1961). On spinodal decomposition. Acta metallurgica.

[20] Devyatko, Y. N. & Tronin, V. N. Uphill diffusion of vacancies and the instability of irradiated materials. *JETP Lett*, **37**(6) (1983).

[21] Nye, J. F. *Physical properties of crystals: their representation by tensors and matrices*. Oxford university press (1985).

[22] Yang L, Dolnik M, Zhabotinsky AM, Epstein IR (2002). Pattern formation arising from interactions between turing and wave instabilities. The Journal of chemical physics.

[23] Evans JH (1971). Observations of a regular void array in high purity molybdenum irradiated with 2 mev nitrogen ions. Nature.

[24] Ipatova I (2017). Radiation-induced void formation and ordering in ta-w alloys. Journal of Nuclear Materials.

[25] Gan J (2014). Microstructural characterization of irradiated u–7mo/al–5si dispersion fuel to high fission density. Journal of Nuclear Materials.

[26] Krishan K (1982). Void ordering in metals during irradiation. Philosophical Magazine A.

[27] Ghoniem NM, Walgraef D, Zinkle SJ (2001). Theory and experiment of nanostructure selforganization in irradiated materials. Journal of computer-aided materials design.

[28] Evans JH (2005). Simulations of the effects of 1-d interstitial diffusion on void lattice formation during irradiation. Philosophical Magazine.

[29] Semenov AA, Woo CH (2011). Interfacial energy in phase-field emulation of void nucleation and growth. Journal of nuclear materials.

[30] Woo CH, Frank W (1985). A theory of void-lattice formation. Journal of Nuclear Materials.

[31] Cahn JW, Hilliard JE (1958). Free energy of a nonuniform system. i. interfacial free energy. The Journal of chemical physics.

[32] Scalapino DJ, Huberman BA (1977). Onset of an inhomogeneous state in a nonequilibrium superconducting film. Physical Review Letters.

[33] Gao, Y. *et al*. Theoretical prediction and atomic kinetic monte carlo simulations of void superlattice self-organization under irradiation. *Scientific reports*, **8** (2018). 10.1038/s41598-018-24754-9 PMC5920090 29700395

[34] Gao, Y. *et al*. Formation and self-organization of void superlattices under irradiation: A phase field study. *Materialia* (2018).

[35] Gaston DR (2015). Physics-based multiscale coupling for full core nuclear reactor simulation. Annals of Nuclear Energy.

[36] Schwen D, Aagesen LK, Peterson JW, Tonks MR (2017). Rapid multiphase-field model development using a modular free energy based approach with automatic differentiation in moose/marmot. Computational Materials Science.

[37] Gibson JB, Goland AN, Milgram M, Vineyard GH (1960). Dynamics of radiation damage. Physical Review.

[38] Trinkaus H, Singh BN, Foreman AJE (1993). Impact of glissile interstitial loop production in cascades on defect accumulation in the transient. Journal of Nuclear Materials.

[39] Pasianot RC, Monti AM, Simonelli G, Savino EJ (2000). Computer simulation of sia migration in bcc and hcp metals. Journal of Nuclear Materials.

[40] Bullough R, Eyre BL, Krishan K (1975). Cascade damage effects on the swelling of irradiated materials. Proc. R. Soc. Lond. A.

[41] Yu H-C, Lu W (2005). Dynamics of the self-assembly of nanovoids and nanobubbles in solids. Acta Materialia.

[42] Hu S, Henager CH (2009). Phase-field modeling of void lattice formation under irradiation. Journal of Nuclear Materials.

[43] Millett PC, Rokkam S, El-Azab A, Tonks M, Wolf D (2009). Void nucleation and growth in irradiated polycrystalline metals: a phase-field model. Modelling and simulation in materials science and engineering.

[44] Jäger W, Trinkaus H (1993). Defect ordering in metals under irradiation. Journal of nuclear materials.

[45] Johnson PB, Thomson RW, Mazey DJ (1990). Large bubble-like features ordered on a macrolattice in helium-implanted gold. Nature.

[46] Johnson PB, Mazey DJ (1995). Gas-bubble superlattice formation in bcc metals. Journal of nuclear materials.

[47] Gan J (2010). Transmission electron microscopy characterization of irradiated u–7mo/al–2si dispersion fuel. Journal of Nuclear Materials.

[48] Gan J (2012). Tem characterization of u–7mo/al–2si dispersion fuel irradiated to intermediate and high fission densities. Journal of Nuclear Materials.

