# Supplementary material for: Bifurcation and Pattern Symmetry Selection in Reaction-Diffusion Systems with Kinetic Anisotropy
Source: Sci Rep. 2019 May 24;9:7835. doi: 10.1038/s41598-019-44303-2 (PMC6534577; doi:10.1038/s41598-019-44303-2)
Supplement: Supplementary file 1 — Supplemental Info [file 41598_2019_44303_MOESM1_ESM.pdf]

Supplemental Information for:
Bifurcation and Pattern Symmetry Selection in Reaction-Diffusion Systems with Kinetic Anisotropy

Yipeng Gao*, Yongfeng Zhang*, Daniel Schwen, Chao Jiang, Jian Gan

Idaho National Laboratory (INL), Idaho Falls, ID 83415, USA

I. DETAILED ANALYSIS OF THE CHARACTERISTIC WAVELENGTH AND SYMMETRY OF SUPERLATTICES

With a linear approximation of the reaction term (Eq. 4), the original kinetic equations (Eqs. 1 & 2) can be represented in Fourier space. Here we focus on the waves with finite wave length ($\mathbf{k} \neq 0$),

$$\frac{\partial \tilde{X}}{\partial t} = \{\nabla \cdot M \nabla \frac{\delta F}{\delta X}\}_{\mathbf{k}} - \sum_{i=1}^{n} K\bar{Y}_i\tilde{X} - \sum_{i=1}^{n} K\bar{X}\tilde{Y}_i \tag{S1}$$

$$\frac{\partial \tilde{Y}_i}{\partial t} = \{\nabla \cdot \mathbf{D}_i \nabla Y_i\}_{\mathbf{k}} - K\bar{X}\tilde{Y}_i - K\bar{Y}_i\tilde{X}, \quad i = 1, 2, ..., n \tag{S2}$$

The total free energy of a non-uniform system can be described as below, with the gradient term incorporated.

$$F = \int [f(X) + \frac{1}{2}\kappa(\nabla X)^2] d^3r \tag{S3}$$

f is the bulk free energy density, and κ is the coefficient of gradient energy, which captures the energetic penalty of inhomogeneity.

The general way to describe a 1D diffusion is through a diffusivity tensor (Eq. 3), and its component form can be written as

$$(D_i)_{pq} = D_0 \cdot m_p^i m_q^i \tag{S4}$$

As a result, the Fourier transform of $\nabla \cdot \mathbf{D}_i \nabla$ is

$$-\sum_{p,q} (D_i)_{pq} k_p k_q = -D_0 \sum_{p,q} m_p^i m_q^i k_p k_q = -D_0(\mathbf{m}^i \cdot \mathbf{k})^2 \tag{S5}$$

Considering Eqs. S3 and S5, we can express the Fourier transform of Eqs. S1 and S2 as

$$\begin{aligned}\frac{\partial \tilde{X}}{\partial t} &= G_{11}(\mathbf{k})\tilde{X} + \sum_{i=1}^{n} G_{1,i+1} \cdot \tilde{Y}_i \\ &= [-Mf''|\mathbf{k}|^2 - M\kappa|\mathbf{k}|^4 - \sum_{i=1}^{n} K\bar{Y}_i]\tilde{X} - \sum_{i=1}^{n} K\bar{X}\tilde{Y}_i\end{aligned} \tag{S6}$$

$$\begin{aligned}\frac{\partial \tilde{Y}_i}{\partial t} &= G_{i+1,1} \cdot \tilde{X} + G_{i+1,i+1}(\mathbf{k}) \cdot \tilde{Y}_i \\ &= -K\bar{Y}_i\tilde{X} - [D_0(\mathbf{m}^i \cdot \mathbf{k})^2 + K\bar{X}]\tilde{Y}_i, \quad i = 1, 2, ..., n\end{aligned} \tag{S7}$$

f'' is the second-order derivative of f, with respect to X. In the index of G, subscript 1 indicates X, and $2 \sim (n+1)$ indicate n types of Y_i. Only the diagonal terms G_{ii} depends on $\mathbf{k}$. The non-zero off-diagonal terms are $G_{1,i+1} = -K\bar{X}$ and $G_{i+1,1} = -K\bar{Y}_i$.

The solution of the above partial differential equations relies on the eigenvalues of the coefficient matrix (G), which depends on $\mathbf{k}$. The eigenvalues of G, $R(\mathbf{k})$, can be determined by

$$[G_{11}-R(\mathbf{k})]\prod_{j=1}^{n}[G_{j+1,j+1}-R(\mathbf{k})]-\sum_{i=1}^{n}\frac{G_{1,i+1}G_{i+1,1}}{G_{i+1,i+1}}\prod_{j=1}^{n}[G_{j+1,j+1}-R(\mathbf{k})]=0 \tag{S8}$$

For a given $\mathbf{k}$, if there is one eigenvalue larger than zero, the system will lose stability to the X wave with wave vector $\mathbf{k}$, i.e., such a wave will develop. At the critical point that the system starts to lose stability, the largest eigenvalue is a maximum at zero, with respect to $\mathbf{k}$.

For the largest eigenvalue, the critical condition is described by

$$R(\mathbf{k}_c)=0 \tag{S9}$$

$$\frac{dR(\mathbf{k})}{d\mathbf{k}}|_{\mathbf{k}=\mathbf{k}_c}=0 \tag{S10}$$

The above problem for the extremum of eigenvalue (of coefficient matrix G) can be converted to a problem for the extremum of determinant, so that the conditions in determining the critical wave vector $\mathbf{k}_c$ should include the following two equations.

$$det[G(\mathbf{k}_c)]=0 \tag{S11}$$

$$\frac{d\{det[G(\mathbf{k})]\}}{d\mathbf{k}}|_{\mathbf{k}=\mathbf{k}_c}=0 \tag{S12}$$

which can be expressed as below, with all the non-zero terms in G considered.

$$\left\{G_{11}\prod_{j=1}^{n}G_{j+1,j+1}-\sum_{i=1}^{n}\frac{G_{1,i+1}G_{i+1,1}}{G_{i+1,i+1}}\prod_{j=1}^{n}G_{j+1,j+1}\right\}|_{\mathbf{k}=\mathbf{k}_c}=0 \tag{S13}$$

$$\frac{d}{d\mathbf{k}}\left\{G_{11}\prod_{j=1}^{n}G_{j+1,j+1}-\sum_{i=1}^{n}\frac{G_{1,i+1}G_{i+1,1}}{G_{i+1,i+1}}\prod_{j=1}^{n}G_{j+1,j+1}\right\}|_{\mathbf{k}=\mathbf{k}_c}=0 \tag{S14}$$

Note that the solution $\mathbf{k}_c$ corresponds to a minimum of $det(G)$ if n is odd, while it corresponds to a maximum if n is even. For simplicity, we assume all types of Y_i are equivalent (could be due to crystallographic degeneracy), so that $\bar{Y}_i=\bar{Y}$. Here we consider the following cases.

Case (i): There is only one type of Y_i.

Eqs. S13 and S14 are reduced to

$$G_{11}(\mathbf{k}_c)G_{22}(\mathbf{k}_c)-G_{12}G_{21}=0 \tag{S15}$$

$$\frac{d[G_{11}(\mathbf{k})G_{22}(\mathbf{k})-G_{12}G_{21}]}{d\mathbf{k}}|_{\mathbf{k}=\mathbf{k}_c}=0 \tag{S16}$$

Since G_{12} and G_{21} are independent of $\mathbf{k}$, Eq. S16 can be written as

$$\begin{aligned}&(-2Mf''-4M\kappa|\mathbf{k}_c|^2)[D_0(\mathbf{m}^1\cdot\mathbf{k}_c)^2+K\bar{X}]\mathbf{k}_c\\&+(-Mf''|\mathbf{k}_c|^2-M\kappa|\mathbf{k}_c|^4-K\bar{Y})[2D_0(\mathbf{m}^1\cdot\mathbf{k}_c)]\mathbf{m}=0\end{aligned} \tag{S17}$$

It is clear that the solution of the above equation is

$$|\mathbf{k}_c| = \sqrt{\frac{-f''}{2\kappa}} \quad and \quad \mathbf{m}^1 \cdot \mathbf{k}_c = 0 \tag{S18}$$

With Eq. S15 considered, we can obtain $\mathbf{k}_c$.

$$|\mathbf{k}_c| = \left(\frac{2K^2\bar{X}\bar{Y}}{\kappa M}\right)^{1/4} \tag{S19}$$

In this case, there are infinite number of the critical wave vectors perpendicular to $\mathbf{m}^1$ (i.e., if $\mathbf{m}^1$ is a plane normal vector, all the critical wave vectors are in this plane), with the above magnitude.

Case (ii): There are two types of Y_i.

Eqs. S13 and S14 are reduced to

$$G_{11}(\mathbf{k}_c)G_{22}(\mathbf{k}_c)G_{33}(\mathbf{k}_c) - G_{12}G_{21}[G_{22}(\mathbf{k}_c) + G_{33}(\mathbf{k}_c)] = 0 \tag{S20}$$

$$\frac{d\{G_{11}(\mathbf{k})G_{22}(\mathbf{k})G_{33}(\mathbf{k}) - G_{12}G_{21}[G_{22}(\mathbf{k}) + G_{33}(\mathbf{k})]\}}{d\mathbf{k}}|_{\mathbf{k}=\mathbf{k}_c} = 0 \tag{S21}$$

Similar to Case (i), we can get

$$|\mathbf{k}_c| = \sqrt{\frac{-f''}{2\kappa}} \quad and \quad \mathbf{m}^1 \cdot \mathbf{k}_c = 0 \quad and \quad \mathbf{m}^2 \cdot \mathbf{k}_c = 0 \tag{S22}$$

$$|\mathbf{k}_c| = \left(\frac{4K^2\bar{X}\bar{Y}}{\kappa M}\right)^{1/4} \tag{S23}$$

$\mathbf{m}^1$ and $\mathbf{m}^2$ are the 1D diffusion directions for two different types of Y_i. In this case, there is a unique critical wave vector perpendicular to both $\mathbf{m}^1$ and $\mathbf{m}^2$, with the above magnitude.

Case (iii): There are n types of Y_i ($n \geqslant 3$).

Eqs. S13 and S14 can be written as

$$\left[G_{11}(\mathbf{k}_c) - \sum_{i=1}^{n} \frac{G_{12}G_{21}}{G_{i+1,i+1}(\mathbf{k}_c)}\right] \prod_{j=1}^{n} G_{j+1,j+1}(\mathbf{k}_c) = 0 \tag{S24}$$

$$\frac{d}{d\mathbf{k}}\left\{\left[G_{11}(\mathbf{k}) - \sum_{i=1}^{n} \frac{G_{12}G_{21}}{G_{i+1,i+1}(\mathbf{k})}\right] \prod_{j=1}^{n} G_{j+1,j+1}(\mathbf{k})\right\}|_{\mathbf{k}=\mathbf{k}_c} = 0 \tag{S25}$$

Because of the complexity of the above equations, we reduce Eq. S25 to a dimensionless form, which is used in numerical calculations.

$$\frac{d}{d\mathbf{k}}\left\{a_3(-1)^n\left[a_1 + \sum_{i=1}^{n} \frac{1}{(\mathbf{m}^i \cdot \mathbf{k})^2/|\mathbf{k}|^2 + a_2}\right] \prod_{j=1}^{n} [(\mathbf{m}^j \cdot \mathbf{k})^2/|\mathbf{k}|^2 + a_2]\right\}|_{\mathbf{k}=\mathbf{k}_c} = 0 \tag{S26}$$

where

$$a_1 = \frac{D_0|\mathbf{k}|^2(-Mf''|\mathbf{k}|^2 - M\kappa|\mathbf{k}|^4 - nK\bar{Y})}{K^2\bar{X}\bar{Y}} \tag{S27}$$

$$a_2 = \frac{K\bar{X}}{D_0|\mathbf{k}|^2} \tag{S28}$$

$$a_3 = K^2\bar{X}\bar{Y}(D_0|\mathbf{k}|^2)^{n-1} \tag{S29}$$

a_1, a_2 and a_3 only depend on the magnitude of $\mathbf{k}$, i.e., they are independent of the direction of $\mathbf{k}$. The effects of a_1, a_2, a_3 on the symmetry selection of superlattices are presented in the paper.

When the above equations are applied to understand the formation of irradiation-induced void/gas bubble superlattice, a_1, a_2 and a_3 are determined by the interplay of thermodynamic/kinetic properties of the material systems and radiation conditions. a_1 depends on the diffusivity of interstitials (D_0), the mobility of vacancies (M), average concentrations of vacancies and interstitials before modulation occurs ($\bar{X}$ and $\bar{Y}$), chemical free energy (f), surface energy (κ), recombination rate between vacancies and interstitials (K) and the critical wave vector of superlattices ($\mathbf{k}$). a_2 depends on K, $\bar{X}$, D_0 and $\mathbf{k}$. a_3 depends on K, $\bar{X}$, $\bar{Y}$, D_0 and $\mathbf{k}$. According to unit analyses, a_1 and a_2 are determined to be dimensionless, which suggests that they are universal parameters to determine the symmetry selection in such reaction-diffusion systems.

In Case (i) and (ii), the critical vector $\mathbf{k}_c$ can be analytically determined because the direction and magnitude of $\mathbf{k}_c$ could be easily separated in the equations. However, in Case (iii), when the number of Y_i is larger than (or equal to) 3, the direction and magnitude of $\mathbf{k}_c$ are coupled together, so that analytical solution cannot be obtained. Instead, we have to adapt numerical calculations based on the values of a_1, a_2 and a_3. It can be found that the direction of $\mathbf{k}_c$ is determined by $a_1/a_2/a_3$, while $a_1/a_2/a_3$ are functions of $\mathbf{k}_c$ magnitude. The coupling between the direction and magnitude of $\mathbf{k}_c$ is also the mathematical origin of the bifurcation phenomenon presented in our paper.

II. PHASE FIELD MODELING OF SUPERLATTICE FORMATION

A phase field model to investigate the superlattice formation is developed based on the open source MOOSE finite element framework. In MOOSE, the phase field kinetic equations (e.g., Cahn-Hilliard equation) are implemented in a general form which is separated from the thermodynamic information. In addition, the spatial discretization is finite-element-based, which can accommodate various kinds of geometries and boundary conditions. Implicit time integration is utilized with adaptive time stepping, employing the preconditioned Jacobian-free Newton Krylov method and utilizing capabilities provided by the libMesh and PETSc libraries. The MOOSE code is open access and guidance is available for downloading, compiling and using the code: https://www.mooseframework.org/. The results in the present paper can be fully reproduced with this version of MOOSE:

commit f6fb066963c59cb24b2f1aea175ee338dc3e40a0 (HEAD, origin/master)

Merge: 20f6c3043f7bb602ed94f219c4a42a708ee1ecc7

Date: Wed Apr 24 2019

The MARMOT code, which is based on MOOSE and is free to access, requires a license. Request can be submitted at: https://moose.inl.gov/marmot/SitePages/Access.aspx.

Without loss of generality, a genetic formulation of free energy is considered, which suggests an intrinsic thermodynamic instability of the system.

$$f(X) = X^2(1-X)^2 \tag{S30}$$

We use a $100 \times 100 \times 100$ simulation cell, which can capture the formation of FCC and BCC superlattices with given input parameters. Note that the critical wavelength of the superlattice is determined by the input parameters. A smaller simulation cell is also acceptable, if the critical wave length is smaller. Periodic boundary conditions are applied to field variables, X and Y_i. Initially, it is a uniform system with $X = Y_i = 0$. All simulations are performed using the Falcon supercomputing system at Idaho National Laboratory, with 36 cores, 128G Random Access Memory and 60 hours. The dimensionless parameters used in phase field simulations are listed in Table S1. Note that phase field is a deterministic method, and there is no uncertainty in calculations beside the intrinsic rounding error when a double precision float is stored in the computer memory. The critical condition for a perfect superlattice formation is described by the solid lines (e.g., blue line for BCC, yellow line for FCC) in Fig. 1. If a_1 and a_2 gradually change their values deviating from this line, the ordering of the superlattices will become worse and worse, so there is no sharp boundary between lattice and non-lattice regions.

To further confirm the symmetry of superlattices, we plot the superlattice configurations in different viewing directions in Figs. S1-S3, corresponding to Figs. 2(a)-(c) in the paper, respectively.

TABLE S1: Parameters used in phase field simulations

Example	P_X	P_i	K	M	D	κ	1D diffusion
Figs. 2(a),3,4,S1	13.6	2.267	160	300	10000	0.5	$\langle 110 \rangle$
Figs. 2(b),5,S2	13.6	3.400	160	300	10000	0.5	$\langle 111 \rangle$
Figs. 2(c),6,S3	14.7	3.675	204	300	260	0.5	$\langle 111 \rangle$

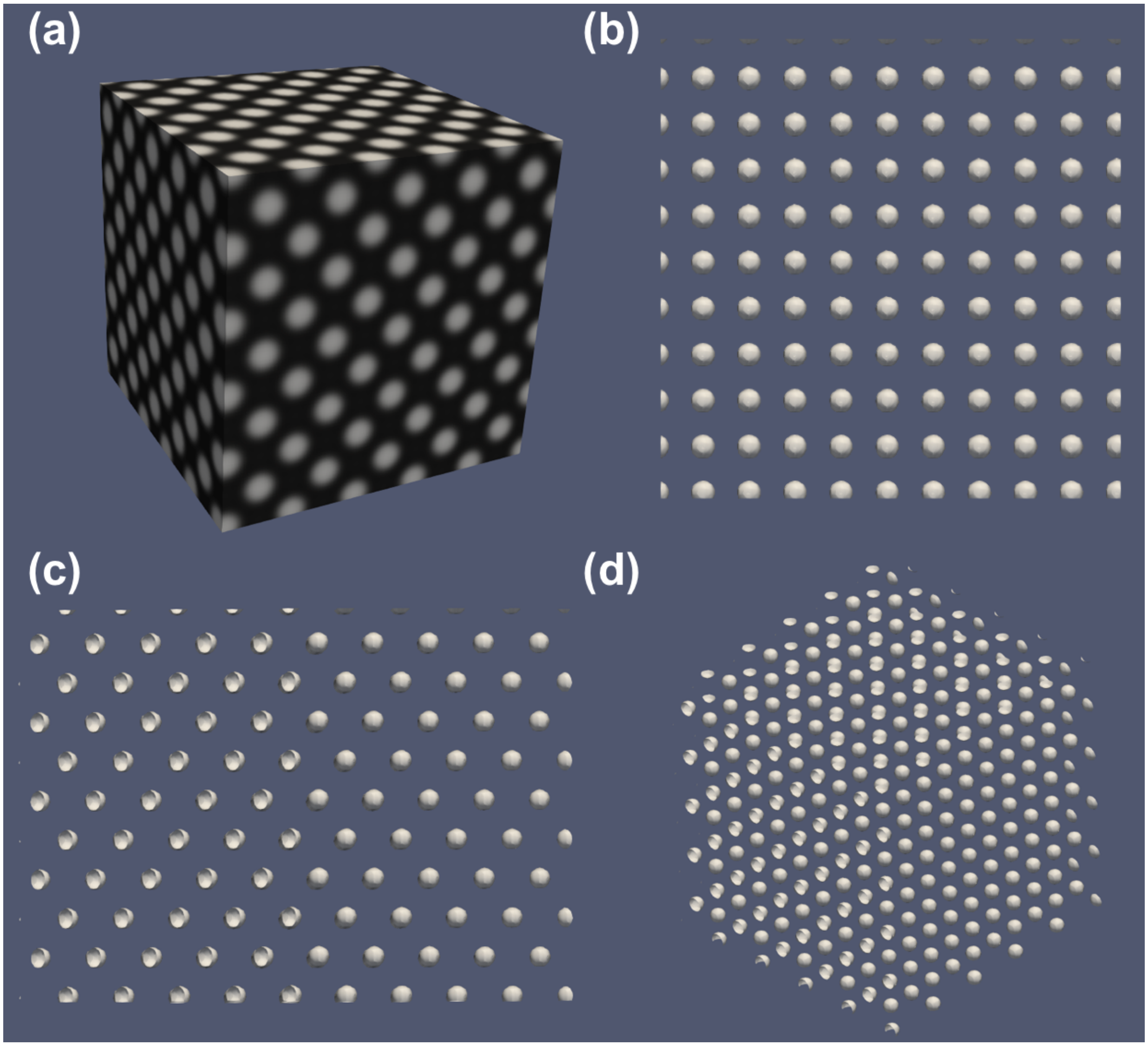

FIG. S1: Phase field simulation results of an FCC superlattice associated with $\langle 110 \rangle$ diffusion. Viewing directions: (b) [100]; (c) [110]; (d) [111].

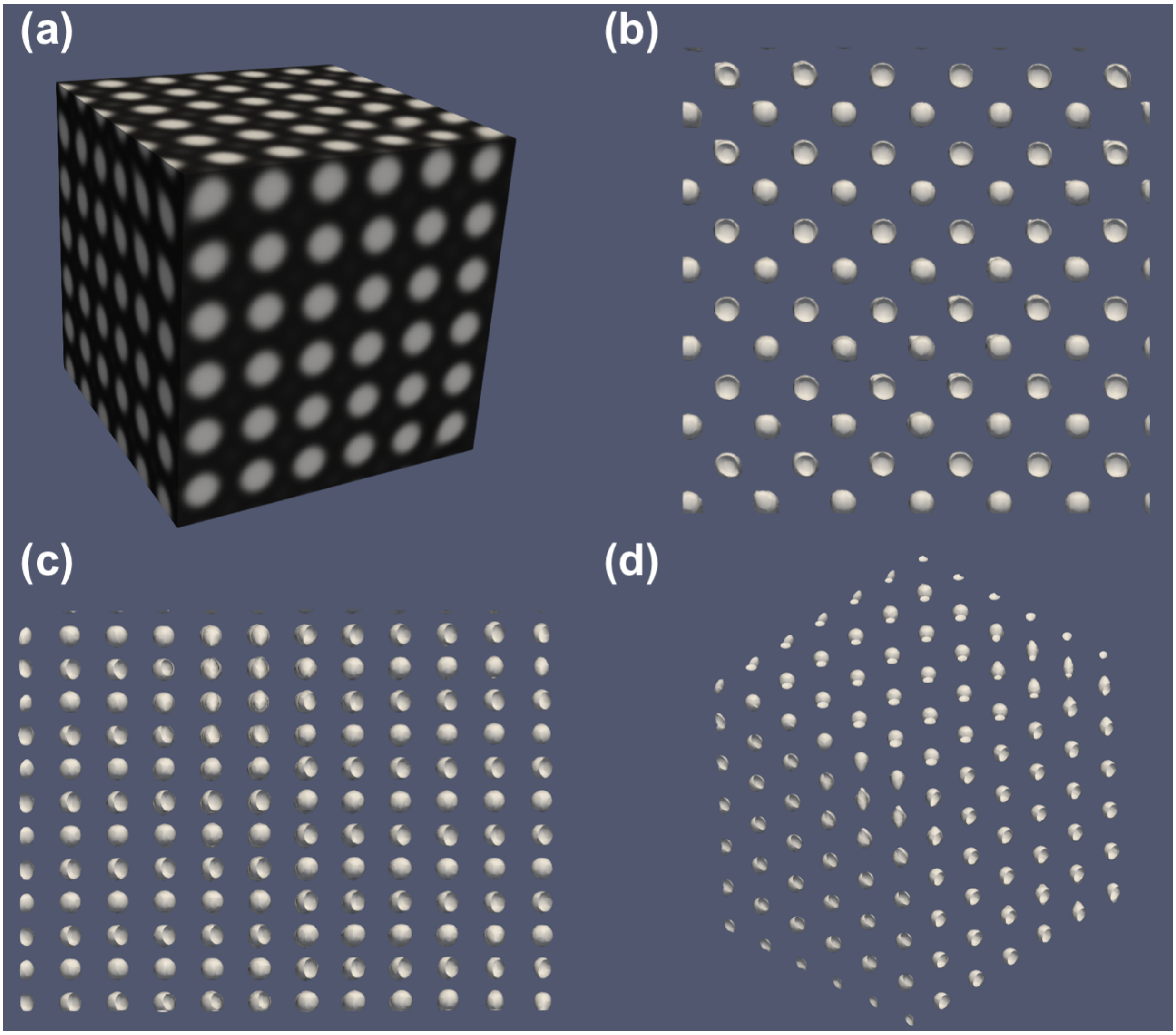

FIG. S2: Phase field simulation results of a BCC superlattice associated with $\langle 111 \rangle$ diffusion. Viewing directions: (b) [100]; (c) [110]; (d) [111].

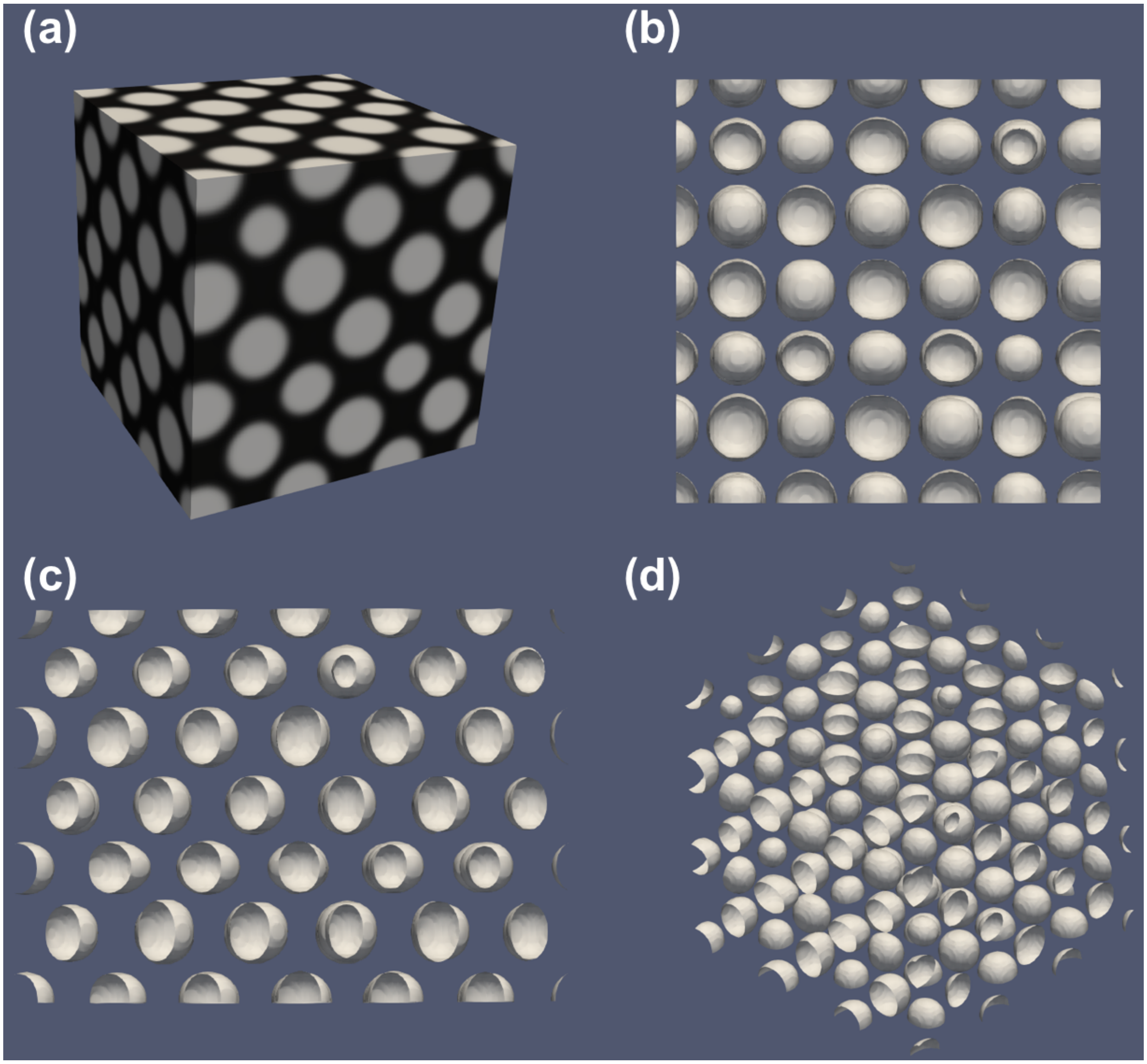

FIG. S3: Phase field simulation results of an FCC superlattice associated with ⟨111⟩ diffusion. Viewing directions: (b) [100]; (c) [110]; (d) [111].
