# Supplementary figures and images for: Bifurcation and Pattern Symmetry Selection in Reaction-Diffusion Systems with Kinetic Anisotropy
Source: Sci Rep. 2019 May 24;9:7835. doi: 10.1038/s41598-019-44303-2 (PMC6534577; doi:10.1038/s41598-019-44303-2)

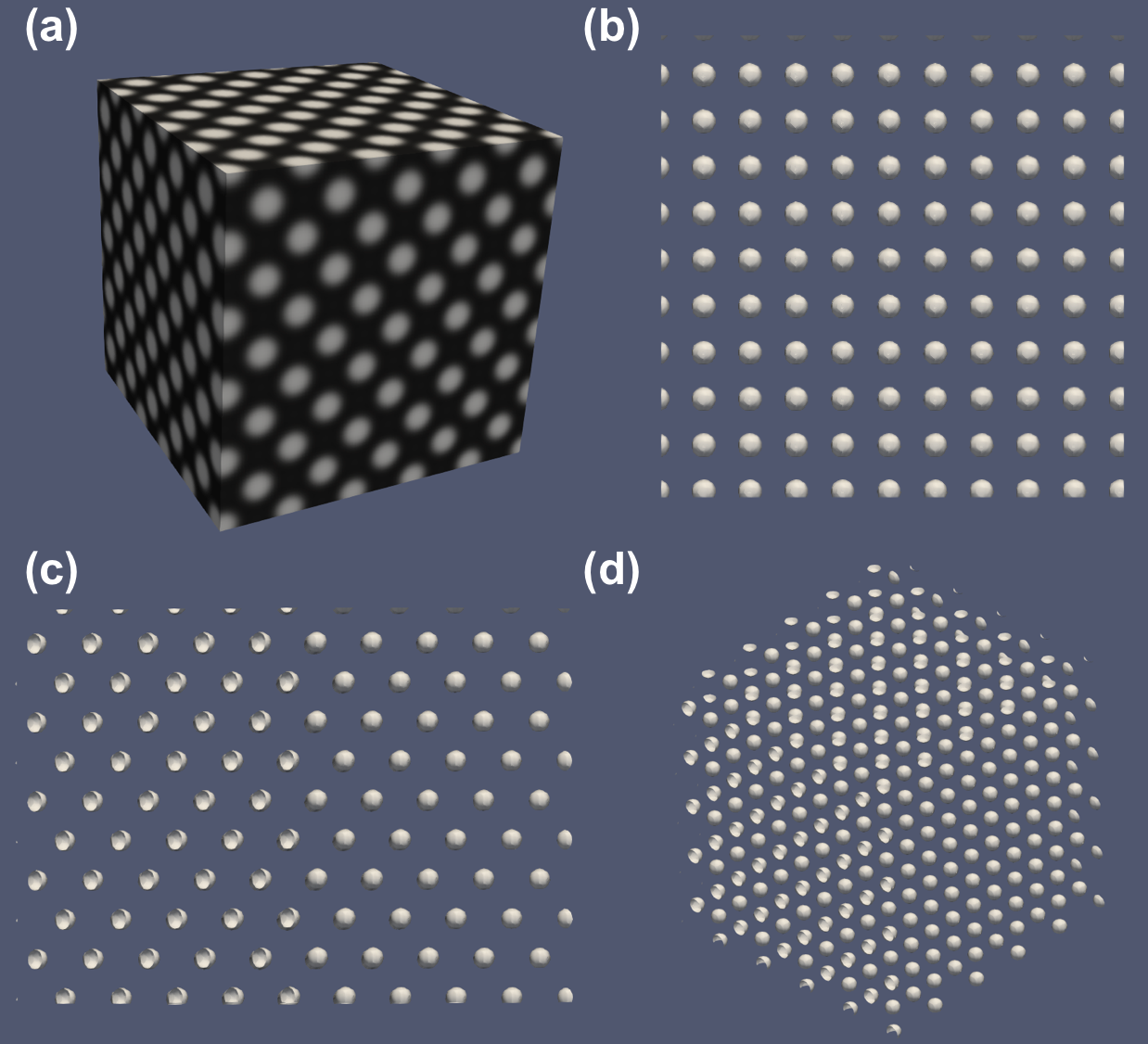

Supplement: Supplementary file 2 — SI LaTeX File [file 41598_2019_44303_MOESM2_ESM.zip › figS1.png]

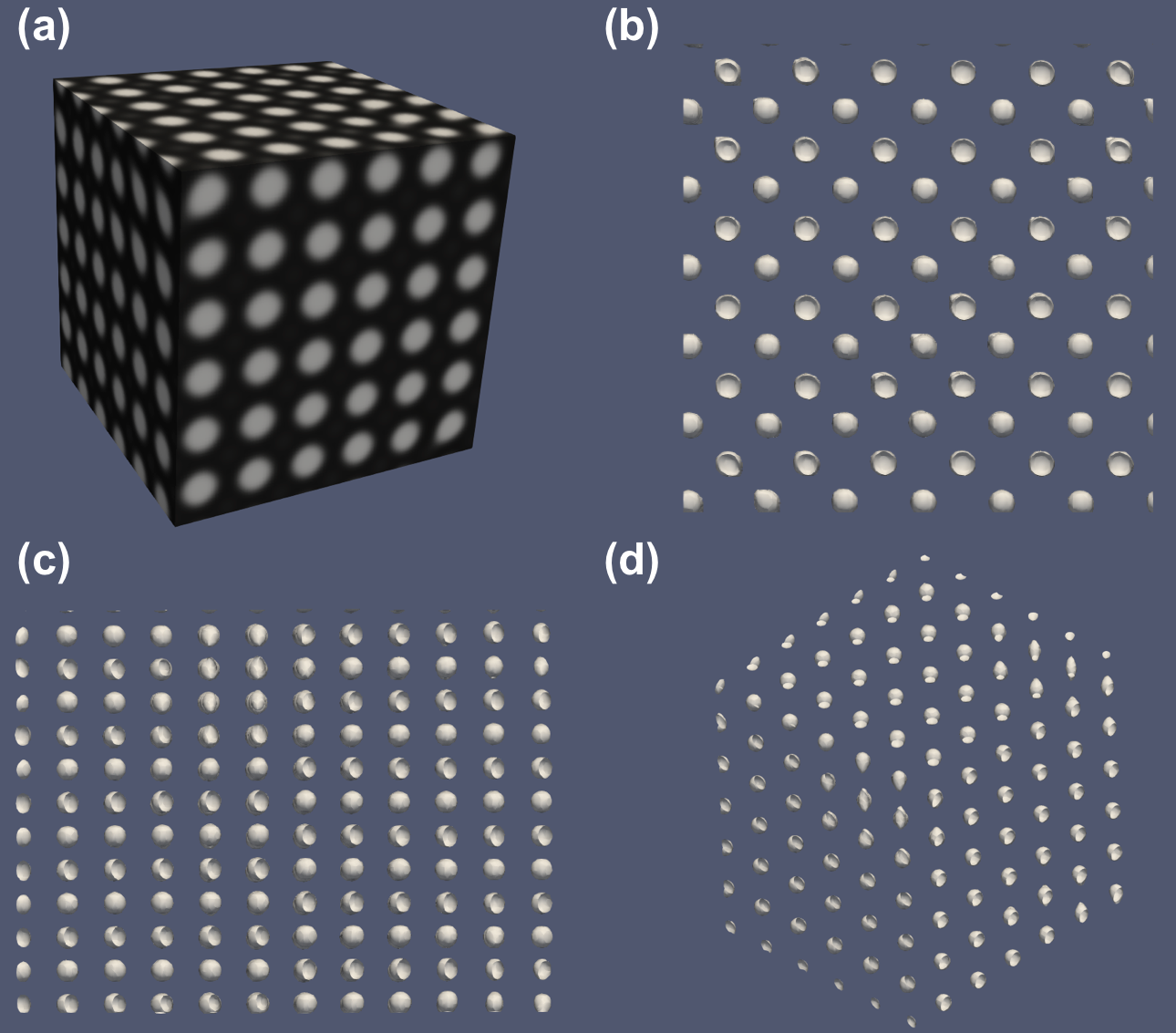

Supplement: Supplementary file 2 — SI LaTeX File [file 41598_2019_44303_MOESM2_ESM.zip › figS2.png]

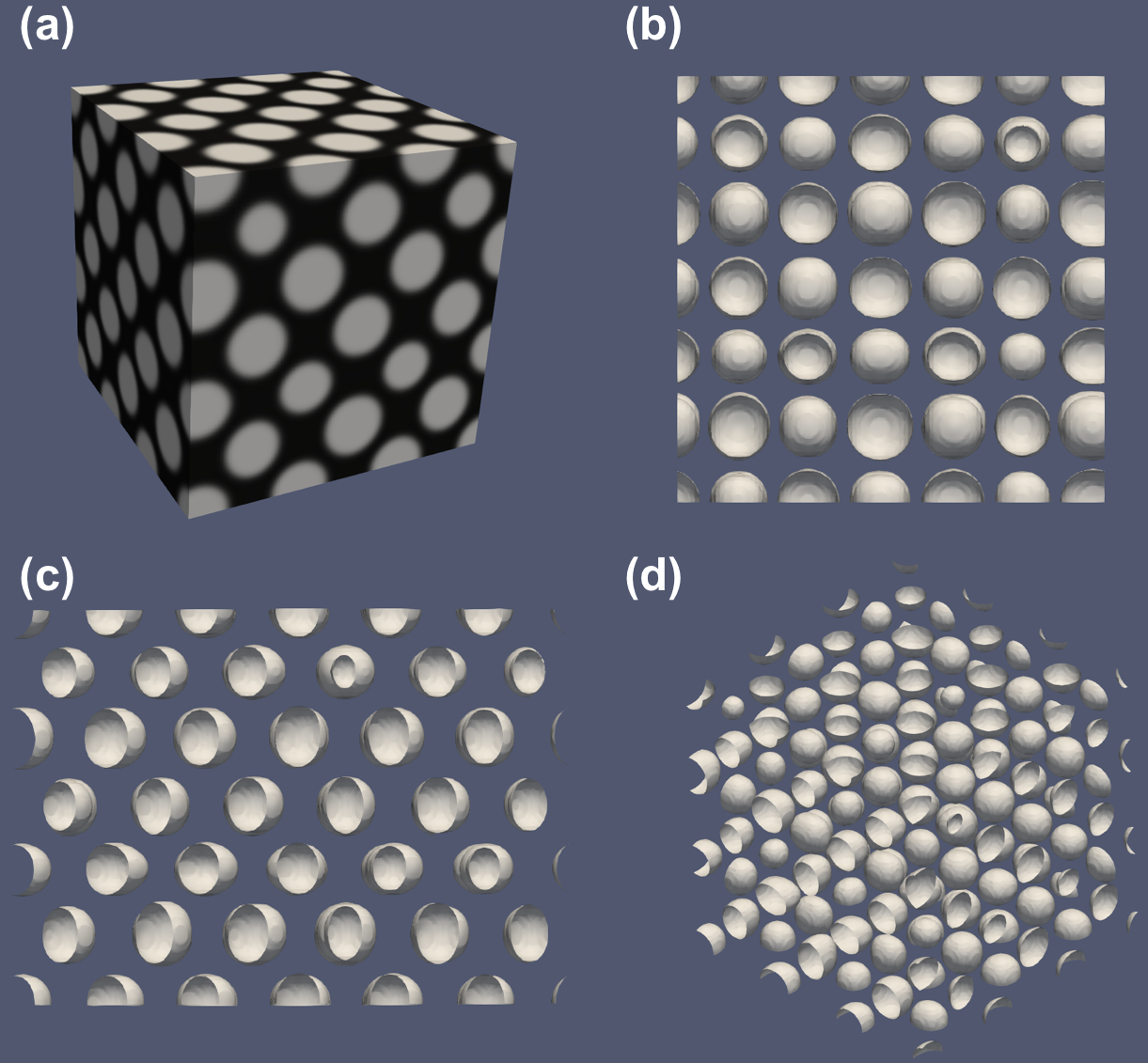

Supplement: Supplementary file 2 — SI LaTeX File [file 41598_2019_44303_MOESM2_ESM.zip › figS3.png]
